# How to target vascular leakage in retinopathy: could a lipid tighten the pipes?

**DOI:** 10.15252/emmm.202317520

**Published:** 2023-03-28

**Authors:** M Luisa Iruela‐Arispe

**Affiliations:** ^1^ Department of Cell and Development Biology, Feinberg School of Medicine Northwestern University Chicago IL USA

**Keywords:** Vascular Biology & Angiogenesis

## Abstract

Retinopathy is one of the more severe complications associated with diabetes. Targeting vascular pathology has shown benefits, but current therapies are costly and have limitations. In this issue of *EMBO Molecular Medicine*, Niaudet *et al* report that the activation of the S1PR1 receptor in endothelial cells is able to block abnormal permeability, neovascular tuft development, and resolve pathological vascular lesions associated with hypoxia‐driven retinopathy.

## Graphical abstract

L. Iruela‐Arispe discusses the manuscript by Niaudet *et al*, in this issue of *EMBO Mol Med*, that reports that the activation of S1PR1 in endothelial cells can block abnormal permeability, neovascular tuft development, and resolve pathological vascular lesions associated with hypoxia‐driven retinopathy.

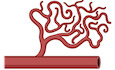

Vascular pathologies of the eye: diabetic retinopathy, age‐related vascular degeneration, and retinopathy of prematurity share similar triggering mechanisms. These include alterations in vascular barrier function that promote leakage of plasma, increases in vascular endothelial growth factor (VEGF), and abnormal proliferation of blood vessels. In recent years, the development of antibodies against VEGF has revolutionized treatment options for these conditions and shows efficacy in preventing loss of vision (Virgili *et al*, 2018). Nonetheless, anti‐VEGF therapy is not recommended during pregnancy or for children. Furthermore, a small percentage of patients experience retinal detachment (anti‐VEGF crunch syndrome) following therapy for proliferative diabetic retinopathy (Tan *et al*, 2021). Thus, despite its multiple benefits, the outlined limitations of anti‐VEGF therapy, associated with the discomfort and technical drawbacks of monthly intravitreal injection, call for the development of new strategies that could be superior to the current first‐line therapy.

At the cellular level, the triggering event in these retinopathies are leaky blood vessels. Under normal conditions, the vasculature of the eye is extremely tight (nonleaky) and designed to avoid loss of plasma from the circulation into the retinal tissue, protecting it from fluid ingress. Junctional complexes between endothelial cells in retinal vessels, similarly to those in the brain, are further reinforced by a basement membrane, and by associated pericytes and astrocytes. In the context of hyperglycemia, molecular alterations in the endothelium and in pericytes alter junctional stability resulting in increased permeability (Fig 1). This is followed by ischemia, which increases VEGF production and further exacerbates barrier instability, promoting endothelial proliferation with abnormal vascular tufts (Antonetti *et al*, 2021). Understanding the mechanisms that maintain the endothelial barrier in the context of pathological stressors is the first step toward the development of new therapeutic approaches for retinopathies.

**Figure 1:**
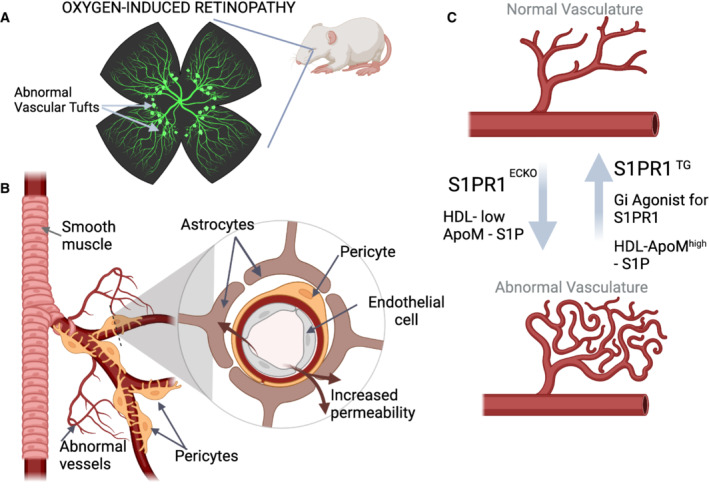
Abnormalities in the vascular barrier underlie retinopathy. Manipulation of the S1P pathway can ameliorate pathology (A) Oxygen‐induced retinopathy in neonatal mouse models results in abnormal vascular tufts in the retina. (B) The progression of the disease is triggered by junctional abnormalities that destabilize association of pericytes with endothelial cells and also alter the endothelial barrier increasing permeability (insert). (C) Using this model, Niaudet *et al* (2023) tested the effect of modulating S1PR1 to ameliorate resulting vascular pathologies. Both genetic (S1PR1ECKO) and pharmacological approaches (HDL‐low‐ ApoM) that reduce S1P signaling worsen the vascular lesions. In contrast, high levels of S1PR1 or increase agonists (small molecules, Gi agonist; or HDL‐ApoM‐S1P) suppressed permeability and vascular tufts. Images generated with BioRender.

Several signaling pathways contribute to endothelial barrier stabilization. Among the most prominent are Notch (Mack *et al*, 2017); Wnt (Ji *et al*, 2021); and the sphingosine‐1‐phosphate (S1P) pathways (Cartier & Hla, 2019). S1P is a lysophospholipid resulting from the metabolism of cell membrane sphingolipids found circulating in association with small and dense HDL particles. Generation of S1P requires the activity of sphingosine kinases (SPHK1 and 2) that catalyze the formation of S1P from sphingosine by phosphorylation (Cartier & Hla, 2019). S1P signals by binding to five G‐protein‐coupled receptors (S1PR1‐5) and in so doing, it regulates myriad of developmental, physiological, and pathological processes (Cartier & Hla, 2019). Of importance, S1PR1 is highly expressed by endothelial cells, and it has been shown to induce adherent junction assembly and promote endothelial–pericyte interactions during development (Paik *et al*, 2004). Furthermore, the activation of the pathway suppresses pathological angiogenesis associated with tumors by stabilizing the vasculature through increasing investment of mural cells and improving junctional complexes (Cartier *et al*, 2020). In relation to retinal vascular retinopathy, plasma levels of S1P have been shown to be inversely correlated with severity of disease (Nilsson *et al*, 2021). This finding, along with those from genetic loss‐ and gain‐of‐function studies in tumor angiogenesis models, support the notion that S1P signaling in the endothelial compartment may be protective against formation and progression of retinopathy.

Through a comprehensive series of approaches to manipulate S1P signaling, Niaudet *et al* (2023) explored the impact of this pathway in the amelioration of oxygen‐induced vascular retinopathy (Niaudet *et al*, 2023). Initially, the team used endothelial‐specific loss‐ and gain‐of‐function of S1PR1. These findings demonstrated that increasing S1PR1 was associated with a reduction in vascular tufts, decreased plasma leakage, and improvement in barrier function (Fig 1). In addition, the group deployed pharmacological means to activate the pathway. Both S1P bound to ApoM‐Fc and a small molecule Gi‐biased S1PR1 agonist suppressed pathological neovascularization in oxygen‐induced retinopathy. An important aspect of this work is that the protective effect noted occurred despite prevalent levels of VEGF. Thus, the mechanism overrides the pervasive complication of elevated VEGF without directly antagonizing the growth factor. Taken together, the data support the concept that activation of the S1P pathway could be beneficial in the treatment of retinopathy associated with vascular dysfunction and offer an important alternative or complementation to anti‐VEGF therapy. In fact, if equivalent in efficacy, S1P activation therapy could bring multiple advantages over VEGF suppression: from improvements on the route of delivery to intervention at an earlier disease stage. However, much work still needs to be done to refine the approach for targeting activation of the pathway prevalently to the endothelium and to understand and limit potential side‐effects emerging from other cell types that also express this receptor. In addition, exploring the best therapeutic modality to activate the pathway should also be carefully considered.

Delivery of S1P using a physiological carrier may offer a valuable approach. Albumin and ApoM act as chaperones to S1P in the circulation. Specifically, ApoM associated with HDL is known to transport approximately 65% of plasma S1P and in this form, S1P can activate its receptor (Christoffersen *et al*, 2011). Following this logic, Niaudet *et al* (2023) found that ApoM was an effective delivery strategy for S1P in their preclinical model. Nonetheless, safety and efficacy should be further explored. Concerns with this mode of delivery relate to the fact that HDL composition and functionality can be modified and become dysfunctional (Márquez *et al*, 2020). For example, glycated HDL fails to bind to S1P and our knowledge of diabetic modifications of HDL‐ApoM‐S1P is limited (Kobayashi *et al*, 2020). Furthermore, oxidation of HDL might also occur and become pathogenic, particularly during inflammatory conditions (Márquez *et al*, 2020). Thus, significant research on the potential for utilizing HDL‐ApoM as carriers should be undertaken. As alternatives, small molecule agonists for S1PR1 could be efficacious. However, once again, formulations and dosage, should be carefully evaluated as S1PR1 agonists have been shown to induce bradycardia and at high doses have deleterious effects on inflammatory cells. In fact, in addition to affecting vascular stability, the S1P‐S1PR pathway regulates the egress of lymphocytes from the spleen and lymph nodes into the circulation. Thus, systemic delivery of any agonist is likely to impact functions of the pathway in a variety of cell types.

Importantly and in relation to the multiple effects of S1P in the immune system, an armamentarium of small molecule antagonists has been developed for treatments that span from multiple sclerosis to inflammatory bowel disease. Some of these induce internalization and degradation of S1PR1 that would deleteriously impact the vasculature by promoting systemic endothelial damage. These adverse effects should be closely monitored.

In summary, Niaudet *et al* (2023) offer an important proof‐of‐concept that highlights S1PR1 activity in the prevention of retinopathy. This work will most certainly pave the way to explore the full spectrum of approaches to best target this multifunctional and complex signaling pathway in pathological settings.

## Author contributions

**M Luisa Iruela‐Arispe:** Conceptualization; supervision; funding acquisition; visualization; writing – original draft; writing – review and editing.
